# Nitrogen deficiency tolerance conferred by introgression of a QTL derived from wild emmer into bread wheat

**DOI:** 10.1007/s00122-024-04692-z

**Published:** 2024-07-17

**Authors:** Nikolai Govta, Andrii Fatiukha, Liubov Govta, Curtis Pozniak, Assaf Distelfeld, Tzion Fahima, Diane M. Beckles, Tamar Krugman

**Affiliations:** 1 Wild Cereal Gene Bank, Institute of Evolution, University of Haifa, Abba Khoushy Ave 199, 3498838 Haifa, Israel; 2 Department of Evolutionary and Environmental Biology, University of Haifa, Abba Khoushy Ave 199, 3498838 Haifa, Israel; 3 Crop Development Centre and Department of Plant Sciences, University of Saskatchewan, Saskatoon, Canada; 4 Department of Plant Sciences, University of California, Davis, CA 95616 USA

## Abstract

**Key message:**

Genetic dissection of a QTL from wild emmer wheat, *QGpc.huj.uh-5B.2,* introgressed into bread wheat, identified candidate genes associated with tolerance to nitrogen deficiency, and potentially useful for improving nitrogen-use efficiency.

**Abstract:**

Nitrogen (N) is an important macronutrient critical to wheat growth and development; its deficiency is one of the main factors causing reductions in grain yield and quality. N availability is significantly affected by drought or flooding, that are dependent on additional factors including soil type or duration and severity of stress. In a previous study, we identified a high grain protein content QTL (*QGpc.huj.uh-5B.2*) derived from the 5B chromosome of wild emmer wheat, that showed a higher proportion of explained variation under water-stress conditions. We hypothesized that this QTL is associated with tolerance to N deficiency as a possible mechanism underlying the higher effect under stress. To validate this hypothesis, we introgressed the QTL into the elite bread wheat var. Ruta, and showed that under N-deficient field conditions the introgression IL99 had a 33% increase in GPC (*p* < 0.05) compared to the recipient parent. Furthermore, evaluation of IL99 response to severe N deficiency (10% N) for 14 days, applied using a semi-hydroponic system under controlled conditions, confirmed its tolerance to N deficiency. Fine-mapping of the QTL resulted in 26 homozygous near-isogenic lines (BC_4_F_5_) segregating to N-deficiency tolerance. The QTL was delimited from − 28.28 to − 1.29 Mb and included 13 candidate genes, most associated with N-stress response*,* N transport, and abiotic stress responses. These genes may improve N-use efficiency under severely N-deficient environments. Our study demonstrates the importance of WEW as a source of novel candidate genes for sustainable improvement in tolerance to N deficiency in wheat.

**Supplementary Information:**

The online version contains supplementary material available at 10.1007/s00122-024-04692-z.

## Introduction

Wheat (*Triticum aestivum* L.) is a major cereal crop, providing 23% of calories and protein in the human diet (Shewry and Hey 2015). Nitrogen (N) is a critical macronutrient that supports wheat growth and development. Factors that limit N in the soil lead to reduced grain yield (GY) and grain protein content (GPC), thereby negatively affecting food security (Tedone et al. 2018; Zuluaga et al. 2018; Fu et al. 2022; Kaur et al. 2022; de Castro et al. 2022). The relationship between carbon (C) and N metabolism in wheat determines yield and GPC, and depends on many factors, including those that play crucial roles in N assimilation and partitioning, e.g., anthesis date, grain-filling duration, and canopy senescence; the cultivar’s genetic background; and the amount and timing of N-fertilizer application (Fatholahi et al. 2020; Wang et al. 2020; Gezahegn et al. 2022). N availability is crucial for it's uptake and utilization by plants, soil structure and environmental conditions, including water availability, are important determinants of this trait. In Mediterranean environments, wheat is mainly grown under rain-fed conditions, and crops often experience water stress. Under such conditions, even if N is available, it will not be taken up. As a result, water scarcity, N deficiency, or a combination thereof, are major causes of yield loss in bread wheat production (De Laporte et al. 2021; Fu et al. 2022; Kaur et al. 2022; Duma et al. 2022). To counteract N deficiency, N can be applied at an early stage of wheat growth; however, this N can be leached from the soil after heavy rains and be lost as a gas, or be immobilized before its uptake by the plant, thus affecting its efficient absorption (Subedi et al. 2007; Zörb et al. 2018; Ghimire et al. 2021). Breeding cultivars for improved water-use efficiency and N-use efficiency (NUE) is therefore, a cost-effective and sustainable approach to increasing GY and GPC in wheat (Ullah et al. 2019).

To meet the growing global demand for food, the use of N fertilizers has increased by 20% over the last 50 years (Zhang et al. 2017; Gu et al. 2023). Wheat cultivation accounts for 18.2% of all N fertilizer used for agronomy (Heffer et al. 2013; Teng et al. 2022), but only 30–40% of the applied N fertilizer is taken up by the plant (Swarbreck et al. 2019; Milner et al. 2022). Inefficient N use leads to water and air pollution, economic losses, and degradation of natural resources; therefore, optimizing the efficiency of N-fertilizer uptake by the plants enable lower N input that would improve agricultural and environmental sustainability (Araus et al. 2020; Fatholahi et al. 2020; Wang et al. 2020; Ghimire et al. 2021; Bharati et al. 2022; Duma et al. 2022; Gezahegn et al. 2022; Effah et al. 2023). Therefore, an understanding of the complexity of regulatory mechanisms controlling NUE, especially when N is limited in the environment, is of great value (Kant et al. 2011; Fan et al. 2019; Hawkesford and Riche 2020; Vishnukiran et al. 2020).

NUE is determined by the ratio of GY to applied N, and the effective utilization of N also influences GPC. It is determined by two processes: N-uptake efficiency (NUpE), which is related to the amount of absorbed N relative to N available in the soil, and N-utilization efficiency (NUtE), measured as yield relative to plant N (Teng et al. 2022; Ayadi et al. 2022). Furthermore, these processes are dependent on the interplay between the genetics of the cultivar, agronomic practices, and environmental conditions, therefore, optimizing these factors will contribute to improved NUE. An approach for introducing genetic diversity of NUE in wheat, especially from landraces or crop wild relatives that are relatively low yielding, is to compare plant performance in different genotypes at low and high N inputs. The idea behind this approach is that traits associated with NUE may only be expressed under low-N conditions (Hawkesford 2017; Fan et al. 2019; Ivić et al. 2021). NUE is influenced by numerous genes which vary among wheat cultivars, some exhibiting better NUtE leading to higher GPC, these include genes associated with N uptake, assimilation, N transport and partitioning (Tegeder 2014; Hawkesford 2017; Wang et al. 2018; Hawkesford and Riche 2020; Alfatih et al. 2020; Rawal et al. 2022; Peng et al. 2022; Zayed et al. 2023). Some of these genes have been found to improve the response to N deficiency in wheat by accumulation of abscisic acid (ABA), especially in guard cells, which leads to stomatal closure (Wilkinson et al. 2007; Seo and Koshiba 2011). This phenomenon is common to plants' adaptive responses to both N starvation and drought stress occurring at an early developmental stage (Kumar et al. 2019).

Identifying QTL regions and subsequent fine-mapping of those regions, is a frequently used approach to identify candidate genes for complex traits, including NUE or GPC. A QTL database for wheat showes that genes found in QTL regions can be involved in metabolic processes, cellular activities, transporters, catalytic functions, and key regulators such as transcription factors, can be regarded as candidate genes for many agronomic traits (Singh et al. 2021). For example, key genes in the GS/glutamate synthase (GOGAT) cycle, critical for N uptake and GPC control, were found in a GPC QTL regions of *durum* wheat (Nigro et al. 2020; Fortunato et al. 2023). The GPC gene *Gpc-B1* is a NAC transcription factor that was identified and subsequently cloned from a genomic region containing the most significant QTL for GPC. This region was derived from wild emmer wheat (WEW) (*Triticum turgidum* ssp. *dicoccoides*, 2*n* = 4*x* = 28, AABB), which is the tetraploid progenitor of cultivated wheat (Distelfeld et al. 2004; Uauy et al. 2006). Cultivars introgressed with the *Gpc-B1* allele have been successfully adopted worldwide (Tabbita et al. 2017), with consistent positive effects on GPC , Fe, and Zn contents, and only minor negative impacts on yield. However, *Gpc-B1* is a NAC transcription factor known to accelerate leaf senescence, and its use can be disadvantageous in high-yielding environments; additional genes are therefore needed to improve GPC. WEW is regarded as a potential source of advantageous alleles for improving resistance to biotic and abiotic stresses and for enhancing key agronomic traits, including NUE and GPC (Chatzav et al. 2010; Millet et al. 2014; Gioia et al. 2015; Huang et al. 2016; Krugman et al. 2021; El Haddad et al. 2021; Nehe et al. 2022; Sandhu et al. 2023).

We previously identified a QTL (*QGpc.huj.uh-5B.2*) that showed a higher proportion of explained variation (PEV = 13%) under water-limited conditions compared to well-watered conditions (PEV = 7%) (Peleg et al. 2009; Fatiukha et al. 2020). Because water scarcity in the soil can negatively affect N availability, we hypothesized that this QTL includes genes associated with N-deficiency tolerance as a possible mechanism underlying the higher PEV under water-limited conditions. N-deficiency tolerance is associated with high NUE, especially in wild plants, and can improve the efficiency of N uptake. Hence, the main objective of our study was to identify candidate genes for N-deficiency tolerance by fine-mapping the QTL. First, we introgressed the QTL into bread wheat using marker-assisted selection (MAS). Next, we validated the higher GPC in IL99 in a N-deficient field, suggesting that N-deficiency tolerance may be involved. We further confirmed that IL99 was tolerant to N deficiency using a reliable and reproducible system that evaluates plant response to severe N deficiency under controlled conditions. Fine mapping of the GPC QTL region delimited its size from − 28.28 to − 1.29 Mb, which included 13 candidate genes for N-deficiency tolerance. Our study confirms that WEW is an important source of novel variation for genes and QTLs that can be used for the improvement of NUE in wheat under unfavorable conditions.

## Materials and methods

### Marker-assisted selection (MAS) of QTL* QGpc.huj.uh-5B.2* and development of a fine mapping population

The QTL *QGpc.huj.uh-5B.2* was identified by QTL mapping based on 150 recombinant inbred lines (RILs) derived from a cross between *Triticum durum* var. Langdon and WEW (genotype G18-16) (Peleg et al. 2009; Fatiukha et al. 2021). One of these RILs (RIL12), carrying the WEW allele of *QGpc.huj.uh-5B.2*, was selected as a donor parent for MAS of GPC. RIL12 was crossed and backcrossed to the Israeli elite bread wheat cv. Ruta as a female parent for three generations as described previously (Merchuk-Ovnat et al. 2016). Molecular validation of the introgression and the MAS procedure was based on SNPs obtained by genotyping Ruta, RIL12, and the parents of the RIL population (WEW G18-16 and *T. durum* var. Langdon) with the Illumina Infinium 15 K Wheat array (TraitGenetics, Gatersleben, Germany). For MAS we selected two SNPs flanking the QTL, and one in the middle of the QTL, which were converted to a set of three Kompetitive allele-specific polymerase chain reaction (KASP) markers, designed using PolyMarker (Ramirez-Gonzalez et al. 2015) (Table S1). Seeds of BC_3_F_2_ plants that were found to be homozygous for the WEW allele in the three SNPs were selected for seed multiplication to BC_3_F_3_ and evaluation of performance and GPC (data not shown). Three BC_3_F_3_ ILs (72, 73, and 99) that displayed better performance were genotyped (in four biological replicates) using the 25 K Wheat array (TraitGenetics). This enabled us to select IL99-2 (further designated as IL99) as a parent for the development of a large fine mapping population. IL99 was backcrossed again as the female parent with Ruta to produce the BC_4_F_1_ population which was increased to BC_4_F_4_ following genotyping in each generation_,_ as previously described (Deblieck et al. 2022). For the fine mapping procedure, we first used the KASP markers that had been used for MAS and then saturated the QTL region with additional KASP markers obtained by genotyping the parental lines. The additional KASP markers were developed with Illumina short-read exome capture sequence data generated by the Whealbi project (http://wheat-urgi.versailles.inra.fr/Projects/Whealbi) using a custom R script and PolyMarker (http://www.polymarker.info) (Table S2).

### Evaluation of introgression line under three environments

The introgression line IL99 and the recurrent parent Ruta were grown in Israel in 2019 (BC_3_F_4_) and in 2020 (BC_3_F_5_) in relatively small plots due to the low number of seeds that are obtained at the early stages of MAS, with sowing density of (250 seeds/m^2^). Plants were grown under three environments, at the experimental fields of the Israeli Ministry of Agriculture, that provided the agronomic management. N was applied as needed for each experimental site, dependent on soil types, expected residual of N in the soil, and environmental conditions: (i) N deficient field in 2019, in the Upper Galilee at Northern Israel (33.170296, 35.581314); the experiment was conducted in small plots (1.5 m^2^) in four repeats; low N fertilizer was applied (40 kg ha^−1^) 60 days after emergence (DAE); the annual precipitation was 680 mm, and was particularly high at young stage growth. (ii) in 2020, in the Western Galilee near Acre (32.930549, 35.106374), in plots of 1.5 × 4 m in five replicates; N management included a split N dose by 100 kg ha^−1^ at pre-sowing and 35 kg ha^−1^ at 60 DAE; the annual precipitation was 607 mm; (iii) in Reim 2020, in the south of Israel (31.377419, 34.477823), in large plots of 1.5 × 6 m in four replicates; N was applied at pre-sowing (80 kg ha^−1^), and 40 DAE (40 kg ha^−1^); this region is characterized as a semi-desert climate and annual precipitation was 220 mm. Low rains at the beginning of the growth season were supplemented with 60 mm irrigation to ensure germination. All three experiments were conducted in a randomized complete block design. In each of the three experiments, grains were harvested by a small dedicated experimental combine to measure total GY (kg/m^2^). In addition, 15 random spikes were selected from individual plants in each plot to measure thousand kernel weight (TKW, g) and GPC (%). GPC was measured in 1.5 g of grain ground in a Laboratory Mill 3310 (Perten Instruments, PerkinElmer, Waltham, MA, USA). The flour was tested for GPC using a Perten Inframatic 9520 NIR Flour Analyzer. Grain protein deviation (GPD) is defined as the standardized residuals of the regression of GPC on GY (Monaghan et al. 2001; Oury and Godin 2007). GPD was proposed as a selection criterion in wheat breeding programs to screen for increased GPC without a concurrent GY reduction. Least squares regressions of GPC on GY were calculated for the three environments (Acre, Reim, and Upper Galilee) using STATISTICA.V10 and (dplyr and ggplot2) library in R. We applied mixed models and random effects models best linear unbiased prediction (BLUP) that account for the variability introduced by the edge effect by treating it as a random effect using the (nlme) library in R. This approach allows for estimating and adjusting the edge effect while simultaneously analyzing the treatment effects. The plant height (PH, cm) was not measured in Reim, therefore, these data are not included in our analysis.

### N stress phenotyping using a semi-hydroponic system

**Fig. 1:**
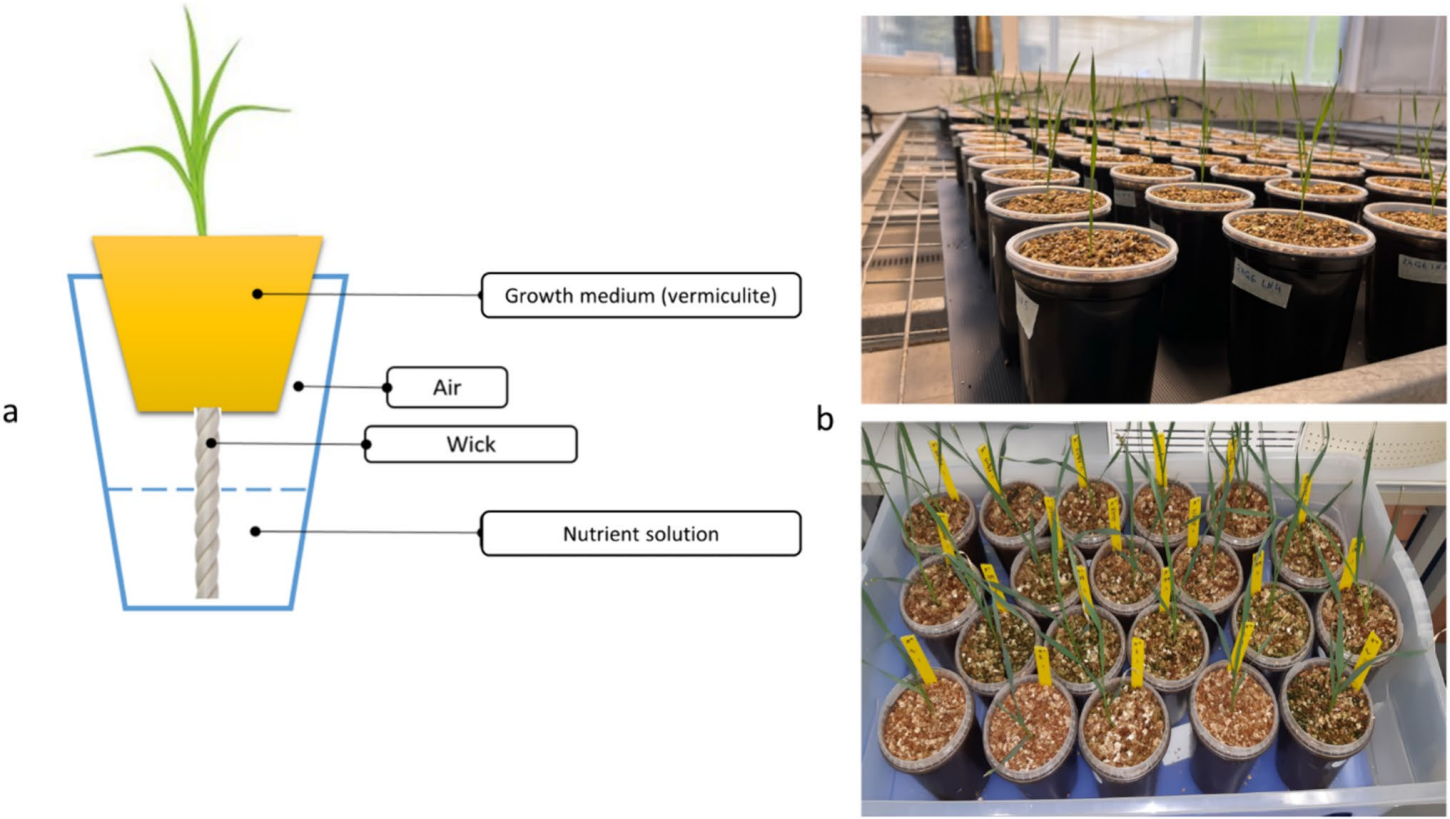
Semi-hydroponics system used for evaluation of wheat growth in LN or FN nutrient solutions at 14 days age. **a** Schematic representation of the system, **b** Image of the system with growing seedlings

N treatments included full N (FN) vs. low N (LN; 10% of the FN) was applied using a semi-hydroponic system that was composed of two pots, one inserted into the other, with a connecting cotton wick transferring the nutrients by a capillary movement (Semananda et al. 2018; Heidari et al. 2022) (Fig. 1).

The top pot (0.5 L) was filled with vermiculite (V2P, granule size: 0.75–2.5 mm) to physically support the plant, and the bottom pot (1.0 L) contained a modified Hoagland’s solution (Hoagland 1920) that was transferred to the top pot by capillary movement. The FN treatment contained: 0.2 mM KH_2_PO_4_, 1 mM MgSO_4·_7H_2_O, 1.5 mM CaCl_2_, 1.5 mM KCl, 0.001 mM H_3_BO_3_, 0.00005 mM (NH_4_)_6_Mo_7_O_24·_4H_2_O, 0.0005 mM CuSO_4·_5H_2_O, 0.001 mM ZnSO_4·_7H_2_O, 0.001 mM MnSO_4·_H_2_O, 0.1 mM FeEDTA, 1.0 mM (NH_4_)_2_SO_4_, and 1 mM KNO_3_; for the LN treatment, the total amount of N was reduced to 10% (0.1 mM (NH_4_)_2_SO_4_ and 0.1 mM KNO_3_). The solutions were maintained at pH 6.0 using 0.1 N H_2_SO_4_.

Seeds were surface sterilized with 70% (v/v) ethanol for 1 min, treated with 0.5% (w/v) sodium hypochlorite for 10 min, and rinsed six times (1 min each time) in sterile distilled water. The seeds were then placed on a wet 11-cm filter paper (VWR®) with clean distilled water and kept sealed in Petri dishes (ø = 90 mm) to g in a growth chamber (Conviron Adaptis CMP6010, Winnipeg, Manitoba, Canada) at 23 °C in the dark. When a coleoptile appeared (Zadoks growth stage 0.7–0.9), healthy and equally developed seedlings were transferred to the semi-hydroponic growing system.

Evaluation of IL99 and Ruta in LN and FN conditions was conducted in four independent experiments. Each experiment included 10 biological replicates of each genotype, tested under LN and FN (each replicate consisted of pot with a single plant). Leaf morphological indices were measured at 14 DAS (Zadoks growth stage 13), including second leaf length (SLL, mm) and number of leaves (NL) as was described for the evaluation of wheat response to abiotic stress at the seedling stage (Sharma et al. 2022). Plants were hand-harvested to measure fresh shoot weight (FSW, mg), and dry shoot weight (DSW, mg) after drying the shoots at 80 °C to constant weight in a heating chamber (Binder ED23 Classic Gravity Convection Oven). FSW indicates the plant's overall growth and water status, which is useful for assessing the effects of water stress, nutrient deficiencies, or other environmental factors. DSW reflects the plant's ability to accumulate dry matter, which is important for evaluating the efficiency of resource utilization. To determine in situ N status in leaves, chlorophyll concentration was measured using a SPAD-502 chlorophyll meter (Konica Minolta, Tokyo, Japan) in the morning from 09^00^ to 10^00^ h. Each value was an average of four measurements recorded from the middle of the leaves. Following the identification of 26 double-homozygous recombinant NILs by KASP markers, we used the semi-hydroponics as a fast-phenotyping system to characterize the response to LN in five biological replicates.

### DNA extraction and KASP genotyping

DNA was extracted in a 96-well plate from fresh leaf tissue of the studied lines following a standard cetyltrimethylammonium bromide (CTAB) protocol slightly modified from (Doyle 1991). DNA quality and purity were assessed by A_260_/A_280_ and A_260_/A_230_ ratios, using a NanoDrop Microvolume Spectrophotometer (Thermo Fisher Scientific, Waltham, MA, USA).  Samples were genotyped using the 25 K SNP array developed by TraitGenetics, and KASP assays were performed in a 96-well plate format (MicroAmp; Applied Biosystems, Waltham, MA, USA) using qPCR (SepOne model, Applied Biosystems). The reaction mixture included 2.5 μL KASP-2 × Master Mix (LGC), 2.2 μL genomic DNA (80–100 ng/µL), and a mix of three KASP primers: 0.08 μL each of primers A and B, and 0.2 μL of primer C per sample (100 ng/µL). qPCR program: hot start at 94 °C for 15 min, followed by 10 landing cycles (94 °C for 20 s; initial landing at 61 °C and decreasing by 1 °C per cycle for 60 s), followed by 30 annealing cycles (94 °C for 60 s; 55 °C for 60 s). Additional cycles were performed to increase the intensity of the fluorescent signals as needed. For each assay, we included a template control (water) and positive heterozygotes of the two parental alleles.

### Candidate gene identification and microcollinearity analysis

Annotation of the genes residing in the *QGpc.huj.uh-5B.2* interval from WEW was based on the *T. dicoccoides* (WEW) genome assembly WEW_v2.1 from https://www.ncbi.nlm.nih.gov/datasets/genome/GCF_002162155.2 (Zhu et al. 2019). We used GeneTribe (https://chenym1.github.io/genetribe/) for homology inference among genetically similar genomes (*T. aestivum* var. Chinese Spring [CS], *T. turgidum* var. Svevo, and *T. dicoccoides* var. Zavitan) that incorporated gene collinearity in the *QGpc.huj.uh-5B.2* region and showed better performance than traditional sequence similarity-based methods in terms of accuracy and scalability (Chen et al. 2020).

### Real-time qPCR

Leaves of the parental lines (Ruta and IL99) and the two double-homozygous recombinant NILs were used for qPCR. Both NILs carry shorter and opposite introgressions from WEW: NIL21 showed resistance to LN and NIL38 was susceptible to LN. Leaf tissues were sampled after 14 days of growth in FN or LN and kept in RNAlater (Thermo Fisher Scientific). RNA extraction was extracted using the RNeasy Plant Mini Kit (Qiagen, Hilden, Germany). Plant of three  biological replicates were used for each N treatment, and each biological replicate consisted of three technical replicates. We selected seven genes located within the *QGpc.huj.uh-5B.2* locus. One gene was included within the fine-mapped QTL (1.29 Mb) region (*UREIDE PERMEASE 1* [*UPS1*]), and six genes were in adjacent regions along the full QTL. The primers used for each gene are listed in Table S3. We used Primer3plus for primer design (https://www.bioinformatics.nl/cgi-bin/primer3plus/primer3plus.cgi). Standard curve method was used to determine qPCR efficiency (Yuan et al. 2008). Acceptance criteria for performance parameters included a dynamic range of four dilutions (1:1, 1:10, 1:100, and 1:1000), a correlation coefficient (R^2^) above 0.96, a PCR efficiency ranging from 90 to 110%, and a respective slope from − 3.6 to − 3.1 (Bustin et al. 2009; Nolan et al. 2013). qPCR results were analyzed by comparative 2^−ΔΔCt^ method as described previously (Livak and Schmittgen 2001). Changes in expression were determined relative to the housekeeping gene *UBIQUITIN*.

## Statistical analysis

All results were analyzed by STATISTICA.V10 (StatSoft Inc. 2011, Tulsa, OK, USA), and R (version 3.4.1). Tukeys test (ANOVA) was used to determine the significance of the difference of each trait with different factors, i.e., genotype (G), environment (E), and the G x E interaction. Statistical significance was set at *p* ≤ 0.05. The correlation analysis assessed the relationships between variables collected during the experiment.

## Results

### Genotyping of introgression lines

We genotyped three BC_3_F_3_ (IL72, IL73, and IL99) to identify the ILs with the shortest introgression and low heterozygosity, which would reduce linkage drag and optimize the fine mapping procedure. Of the 25 K SNPs, we identified ~ 5100 for both the A and B wheat genomes, with an average density of 0.5 SNPs/Mb. After filtering the data and ordering the SNPs based on their physical positions (Table S4), we validated the presence of *QGpc.huj.uh-5B.2* in all three ILs. IL99 had the shortest introgression, which spanned 58.37 Mb – almost tenfold shorter compared to those in IL72 and IL73 (514 Mb). Furthermore, IL99 showed a low level of retained alleles from the original tetraploid parent (LDN) on the short arm of chromosome (Chr) 2A. In contrast, IL72 and IL73 retained additional alleles of LDN or heterozygous regions on Chr 3B, 4B, and 5A. Genotyping also confirmed that none of the three BC_3_F_3_ ILs carried the *Gpc-B1* functional allele from WEW, a major QTL identified in other mapping populations (Uauy et al. 2006; Distelfeld et al. 2007). Taken together, these genotyping results showed that IL99 was the most suitable female parent for backcrossing with Ruta and for developing the fine mapping population.

### Evaluation of the introgression line and *Ruta* in three environments

**Fig. 2:**
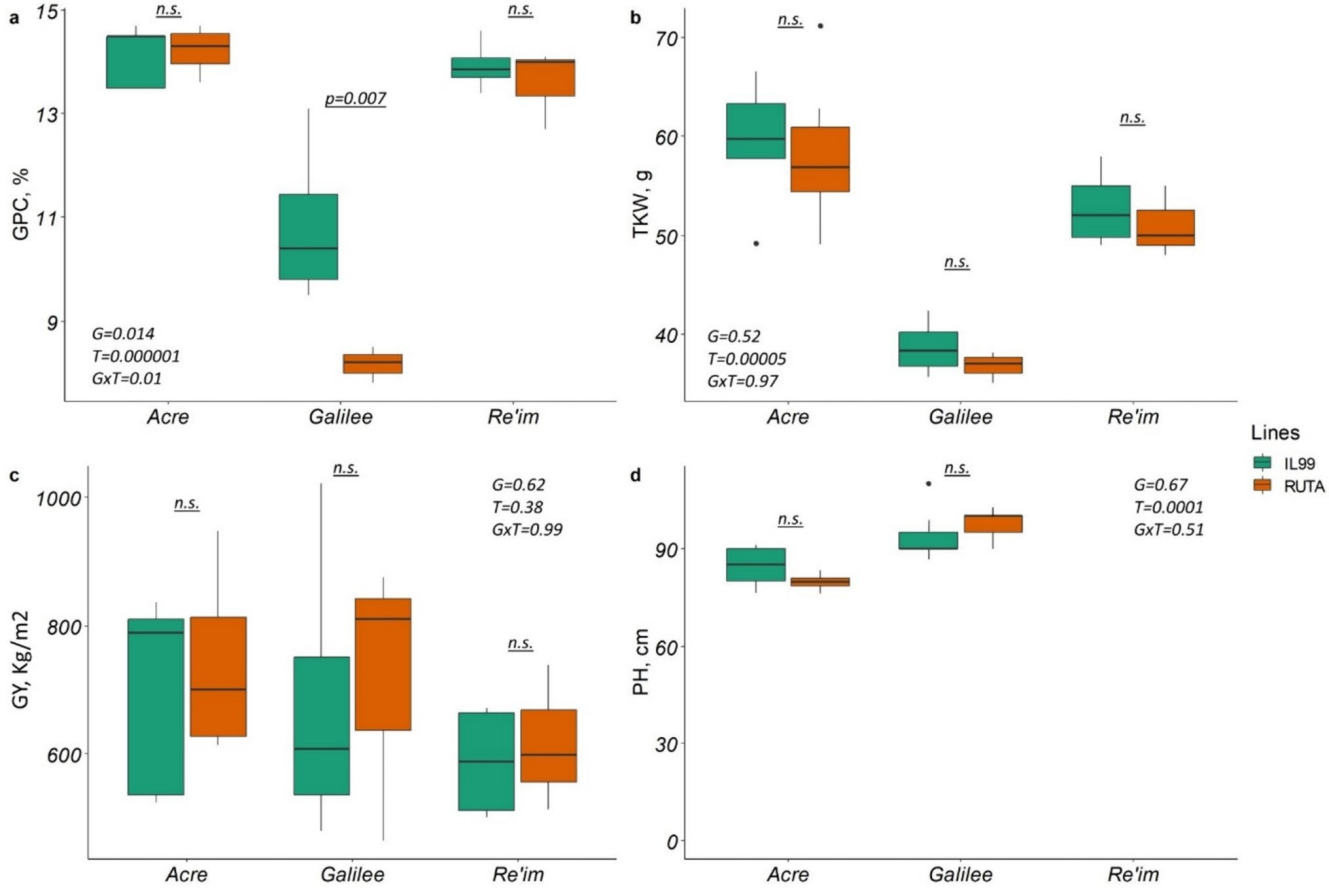
Estimates of the agronomic wheat traits measured in field experiments in 2019–2020. **a** Grain protein content (GPC), **b** Thousand kernel weight (TKW), **c** Grain yield (GY), **d** Plant height (PH). X-axes show the three environments in which the wheat lines were cultivated, i.e., Acre, Reim, and Upper Galilee. The two genotypes, i.e., Ruta and IL99, are indicated by green and red bars, respectively. Each graph shows a different combination of genotype (G), treatment (T), and their interaction (G × T). The *p*-values indicate a significant difference; n.s.—not significant

Our results showed that in the N deficient field, IL99 exhibited higher GPC as33% compared to Ruta (10.84 vs. 8.15, *p* = 0.007). However, no differences in GPC were found between genotypes in the two well-managed N fields. At Acre (14.14 vs. 14.24, *p* > 0.05), and the semi-desert field in Reim, which received 220 mm of rain (13.91 vs. 13.57, *p* > 0.05). Notably, the field at Reim was supplemented with 60 mm of irrigation at the early growth stage to ensure plant germination and establishment. In this field, we found a significant (*p* = 0.045) difference in GPD between the average normalized residues for Ruta (− 0.178) and IL99 (0.0008), indicating that IL99 accumulated higher GPC than predicted from GY alone. In Acre, we detected a significant negative correlation (*r* = − 0.74, *p* < 0.05) between GPC and TKW (Table S6), suggesting a trade-off between GPC and kernel size. However, in the Upper Galilee and Reim, this relationship was not significant, suggesting that other factors were operating. Negative correlations between GPC and TKW are commonly observed when N is not limiting. When N is limited, as in our low N field in Galilee, the plant’s source strength may be reduced, leading to competition for available resources between protein and starch biosynthesis. In such cases, the correlation between GPC and TKW may not be as pronounced or significant (*r* = 0.08, *p* > 0.05). No significant differences were found in GY measured between IL99 and Ruta in two sites, i.e., at Acre of the Upper Galilee (Fig. 2).

The assessment of IL99 and Ruta grown in the three environments showed that GPC and TKW in both genotypes were significantly lower in the N deficient field in the Upper Galilee field, compared to the two other fields that were treated with split N doses. The extremely low GPC values found for both genotypes in Upper Galilee suggest that the field suffered from severe N deficiency that resulted from low fertilization, i.e., a single N treatment of 40 kg ha^−1^, 60) DAE, and possible leaching of N residuals due to heavy rains during the plant establishment period.

### Phenotypic response to LN and FN evaluated using a semi-hydroponic system

**Fig. 3:**
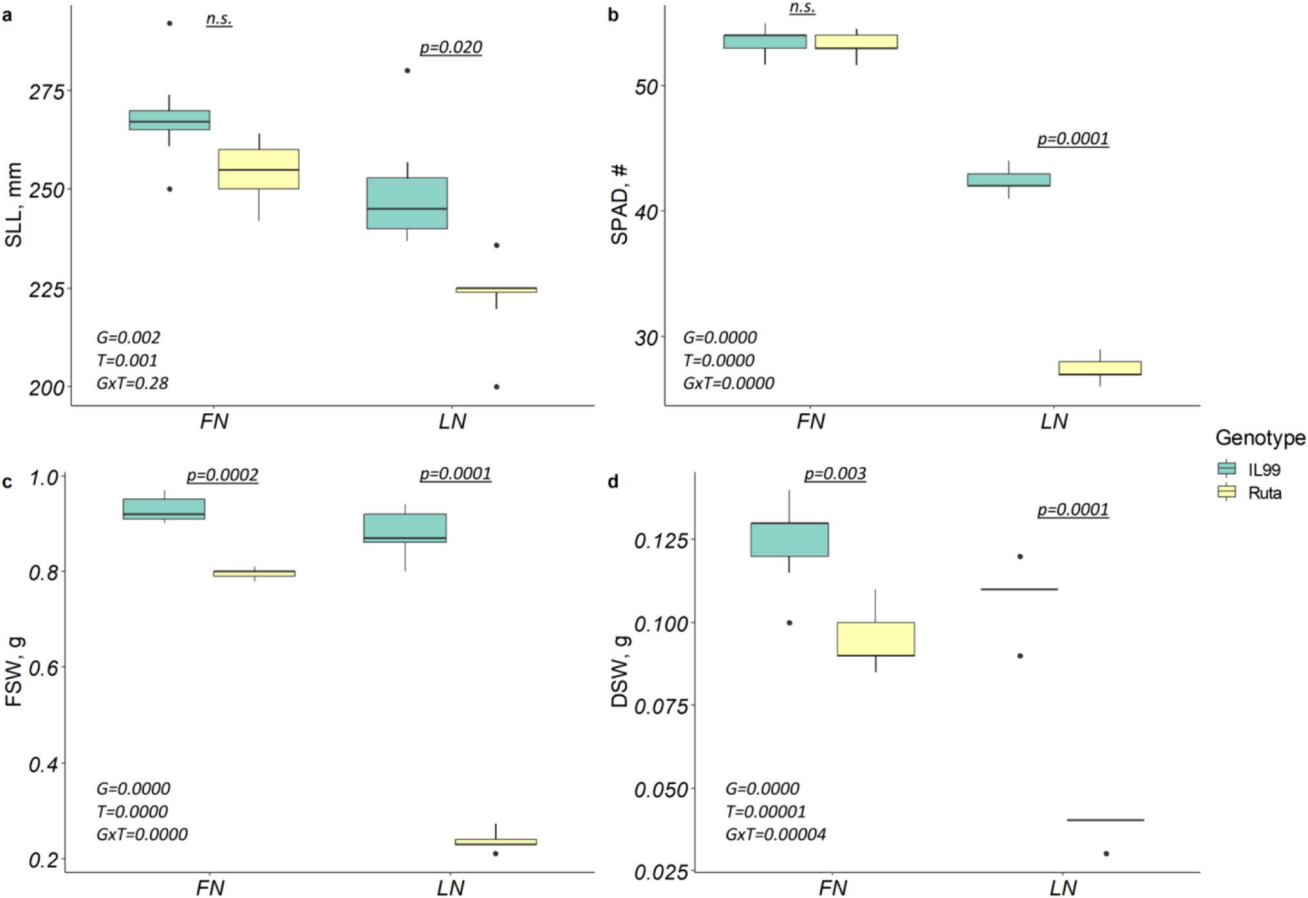
Morphological parameters measured in Ruta and IL99 under LN and FN conditions after 14 days of growth. **a** Second leaf length (SLL), **b** Fresh shoot weight (FSW), **c** Level of chlorophyll accumulation measured by SPAD, **d** Dry shoot weight (DSW). LN – low nitrogen, FN – full nitrogen. The graph presents significant (*p*) and non-significant (n.s.) levels and multivariate tests with different factors: G—genotype; T—treatment, and their interaction (G × T)

To assess LN tolerance in IL99 compared to Ruta and to establish a sensitive, reproducible, and accurate method for phenotyping under controlled conditions, we subjected plants to severe N stress using a semi-hydroponic system. N starvation at the seedling stage can be reliably assessed by monitoring reductions in leaf elongation rates compared with seedlings grown under optimal N (Gioia et al. 2015). Furthermore, measurements of SLL at 14 days were highly correlated with FSW and DSW (*r* = 0.76 and *r* = 0.81, *p* < 0.05) both in Ruta and IL99. SLL, NL, FSW, and DSW did not differ in IL99, or showed only a slight but non-significant change, in response to LN. Therefore, SLL measured after 14 days of N deficiency was used as an indicator to evaluate the response to N stress of recombinants. Our results confirmed that significant changes were found in Ruta in all the measured traits under LN compared to FN, while IL99 showed only small differences, which were not statistically significant for most studied traits (Fig. 3; Table S7).

### Fine mapping of *QGpc.huj.uh-5B.2*

**Fig. 4:**
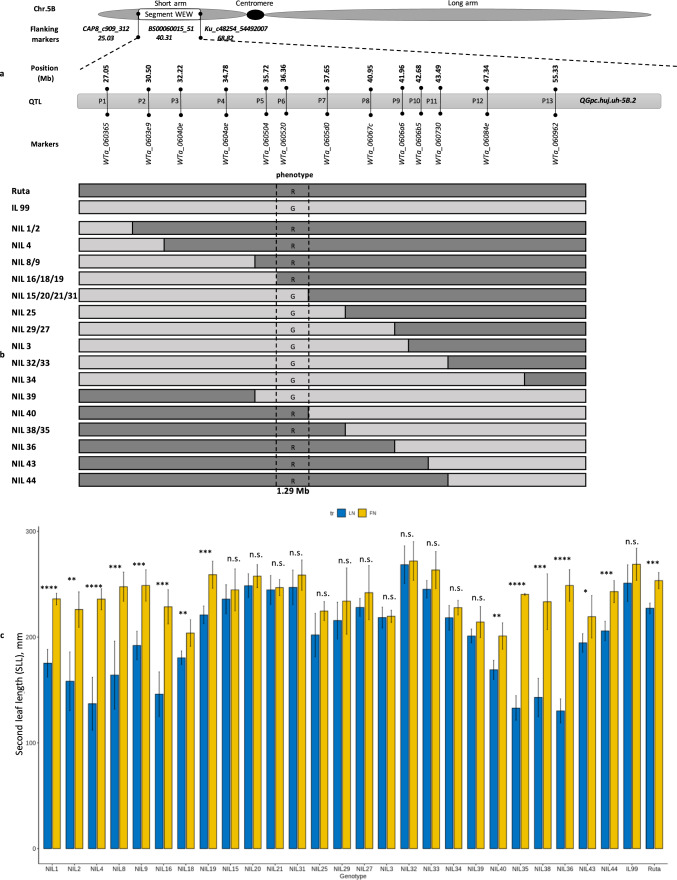
Fine mapping of the *QGpc.huj.uh-5B.2* region by genotyping and phenotyping of 26 NILs. **a** Schematic map of Chr 5B of Ruta, the site of introgression of WEW *QGpc.huj.uh-5B.2*, and below, the position of 13 KASP markers along the QTL (from 27.05 to 55.33 MB), **b** Graphical genotyping and phenotyping of the parental lines Ruta, IL99 and 26 recombinant NILs; *R* allele (Ruta) and* G* allele (IL99), **c** Comparison of the second leaf length (SLL) of 26 NILs and two parental lines grown under FN or LN conditions. (*p < 0.05; **p < 0.01; ***p < 0.0001; ****p < 0.00001); n.s.—not significant

For the fine mapping procedure, we designed 13 KASP markers, two flanking the QTL and 11 saturating the QTL interval, based on SNPs found between the parental lines (LDN and G18-16, RIL12 and Ruta). Exome capture data that produced more than 620,000 SNP/indels at the whole-genome level enabled us to identify 13 SNPs evenly distributed across the QTL region of 28.28 Mb (from 27.05 to 55.33 Mb) (Fig. 4a). We further converted SNPs to KASP markers based on the Chinese Spring RefSeq v2.0 genome (Zhu et al., 2021) (Table S2). IL99 (BC_3_F_4_) was backcrossed with Ruta to produce the BC_4_F_1_ population. To confirm heterozygosity, all BC_4_F_1_ individuals were genotyped using the KASP markers flanking the QTL (P1 WTa_060365, and P13 WTa_060962). To select single- and then double-homozygous recombinants for fine mapping, we first genotyped 1416 BC_4_F_2_ with an additional KASP marker positioned in the middle of the QTL (P6 WTa_060520). This resulted in two types of recombinants at BC_4_F_3_: type I (homozygous at P1, and heterozygous at P6), which were genotyped in the next generation (BC_4_F_4_) with KASP markers P2 to P6; and type II (heterozygous at P6, and homozygous at P13) which were genotyped with KASP markers P6 to P12. In total, we genotyped 2500 BC_4_F_4_ plants using 13 KASP markers along the QTL interval. This procedure enabled us to identify 26 homozygous recombinant NILs, each representing recombination events along the QTL interval (Fig. 4b). These 26 NILs were classified into 16 haplotypes; for example, NILs 15, 20, 21, and 31 each carried an introgression of similar size (Fig. 4b).

**Fig. 5:**
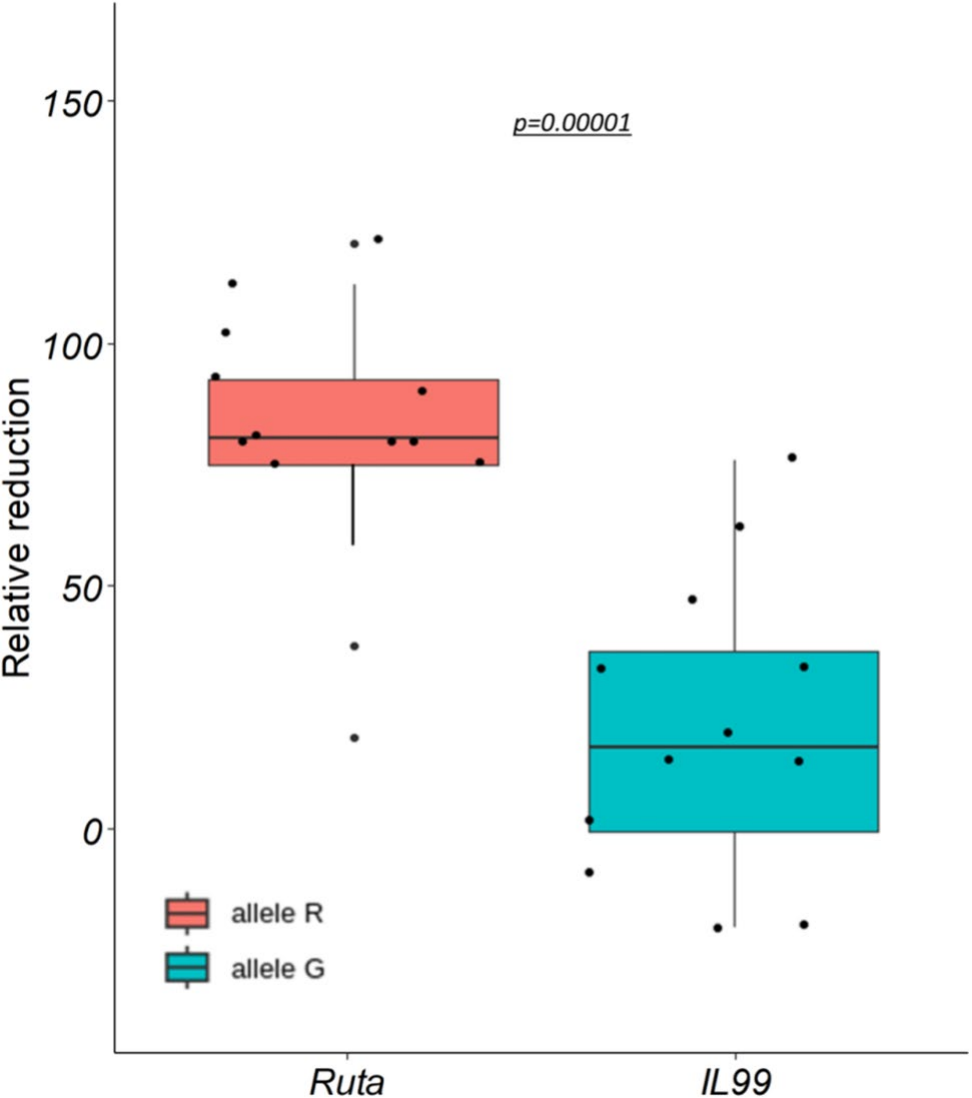
Relative reduction in SLL between LN and FN was identified among the 26 homozygous recombinant NILs. Five biological replicates were used for each NIL having alternative alleles of parental genotypes (*R* and *G*). The *p*-value shows a significant difference between the two alleles

The segregation of phenotypic response to LN found in all 26 BC_4_F_5_ NILs was obtained  using the semi-hydroponic system. The response to LN was recorded by calculating the reduction of SLL between the two conditions. Our results showed that 12 NILs (of 7 haplotype groups) (15, 20, 21, 31, 25, 29, 27, 3, 32, 33, 34, and 39) had no significant reduction in SLL, and were therefore regarded as having an IL99 phenotype (G); and SLL of 14 NILs (1, 2, 4, 8, 9, 16, 18, 19, 40, 35, 38, 36, 43, and 44) was significantly reduced (233.67 vs. 167.78 mm, *p* < 0.05), and regarded as having the Ruta phenotype (R) (Fig. 4c). Comparison of the relative reduction of SLL between the two alleles *R* and *G* (Fig. 5) showed that NILs with the *R* allele exhibit a notable (*p* < 0.05) reduction in leaf growth, with its frequency decreased by approximately 65.8%, whereas NILs of the WEW allele *G* decreased by a smaller margin, around 11.5%. Altogether, the fine mapping which integrated the genotyping and phenotyping of 26 NILs (Fig. 4b) showed that the 12 NILs that were tolerant to N stress shared an introgression of 1.29 Mb (between P6 to P7) with the haplotype of (G) of WEW.

### Candidate genes identification and microcollinearity analysis

The region of 1.29 Mb  (from P6 to P7) was estimated based on the WEW ‘Zavitan’ reference genome. This size differed from the *T. durum* var. Svevo—1.7 Mb (from 37.03 to 38.73), and the CS—1.92 Mb (from 36.6 to 38.52) assemblies. Based on gene annotation of the WEW genome, we identified 13 high-confidence genes (listed in Table 1): two genes (TRIDC5BG005570 and TRIDC5BG005760) of unknown function, and one gene (TRIDC5BG005640) annotated as disease resistance and is probably not associated with N stress. The remaining 10 genes were classified into two categories based on gene ontology (GO) functions or based on available literature information: (1) Five genes were associated with abiotic stress including, TRIDC5BG005540 encoding *pentatricopeptide repeat 336*; TRIDC5BG005560 *encoding a cold-regulated protein*; TRIDC5BG005580 encoding a *histone-lysine N-methyltransferase (HKMT)*; TRIDC5BG005710 encoding *syntaxin-132 (SYP132)*, and TRIDC5BG005770 *metallohydrolase/oxidoreductase (MHO) superfamily protein*. (2) Four genes involved in N transport, N metabolism, and N stress: TRIDC5BG005550 encoding *UPS1;* TRIDC5BG005530 encoding *15-cis-zeta-carotene isomerase (Z-ISO)*; TRIDC5BG00560*0* encoding *importin subunit β1 (KPNB1)* and TRIDC5BG005630 *ATXR6* encoding *serine protease HtrA*.

**Table 1:** Homologs of WEW candidate genes identified in the 1.29 Mb sub-QTL, compared with *T. durum* var. Svevo and CS assemblies. The genes are listed based on their order on the chromosome

#	*T. dicoccoides* Zavitan	Start, bp	End, bp	*T. turgidum* Svevo	*T. aestivum CS*	Gene annotation	GO function
1	TRIDC5BG005520	36,360,061	36,361,911	TRITD5Bv1G013710	TraesCS5B02G033700	*p21-ACTIVATED PROTEIN KINASE-INTERACTING PROTEIN 1*	Signaling pathways/stress
2	TRIDC5BG005530	36,365,122	36,372,548	TRITD5Bv1G013720	NA	*15-cis-ZETA-CAROTENE ISOMERASE*	N stress
3	TRIDC5BG005540	36,396,013	36,398,271	TRITD5Bv1G013670	TraesCS5B02G033500	*PENTATRICOPEPTIDE REPEAT 336*	Stress
4	TRIDC5BG005550	36,404,321	36,406,308	TRITD5Bv1G013770	TraesCS5B02G033400	*UREIDE PERMEASE1*	N utilization and N stress
5	TRIDC5BG005560	36,428,596	36,428,820	TRITD5Bv1G013610	TraesCS5B02G033300	Cold-regulated protein	Cold stress
6	TRIDC5BG005570	36,428,604	36,428,765	NA	NA	Undescribed protein	NA
7	TRIDC5BG005580	36,437,689	36,438,391	TRITD5Bv1G013570	NA	*HISTONE-LYSINE N-METHYLTRANSFERASE ATXR6*	Development and Stress
8	TRIDC5BG005600	36,531,860	36,537,534	TRITD5Bv1G013910	TraesCS5B02G034200	*IMPORTIN SUBUNIT BETA-1*	N stress
9	TRIDC5BG005630	36,820,431	36,822,867	TRITD5Bv1G013990	TraesCS5B02G034400	*SERINE PROTEASE HTRA*	Stress
10	TRIDC5BG005640	36,947,849	36,949,190	TRITD5Bv1G014030	TraesCS5B02G034600	Disease resistance protein	Biotic stress
11	TRIDC5BG005710	37,234,570	37,237,309	NA	TraesCS5B02G035600	*SYNTAXIN-132*	Stress
12	TRIDC5BG005760	37,446,998	37,447,553	TRITD5Bv1G018160	TraesCS5B02G035500	Unknown function	NA
13	TRIDC5BG005770	37,645,630	37,648,660	TRITD5Bv1G014330	TraesCS5B02G035300	*METALLOHYDROLASE/OXIDOREDUCTASE SUPERFAMILY PROTEIN*	Stress

**Fig. 6:**
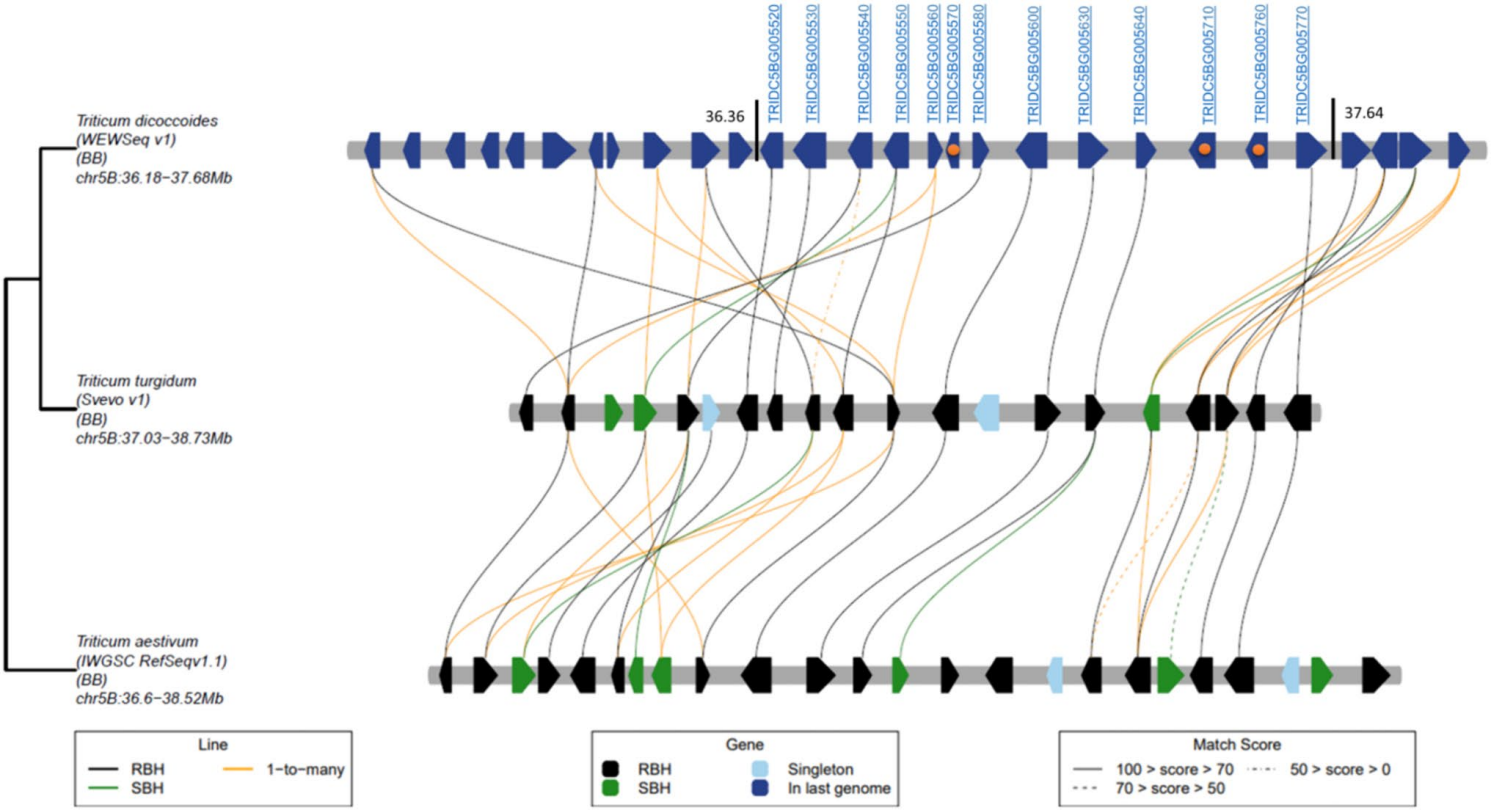
Microcollinearity visualization function showing homologous relationships on a local scale between 13 candidate genes in Zavitan (*T. dicoccoides*), CS (*T. aestivum*), and Svevo (*T. turgidum*). Based on the GeneTribe algorithm, homologous genes are divided into three types: black, green, and yellow lines represent reciprocal best hit (RBH), single-side best hit (SBH), and one-to-many relationships, respectively. The lines represent homologous relationships and are grouped into three groups by score: 0–50, 50–70, and 70–100

Homology indicates sequence similarity due to common ancestry, whereas collinearity refers to homology due to the linear arrangement of genes along a chromosome between genomic regions in the same species (Chen et al. 2020). Microcolinearity analysis of the 1.29 Mb sub-QTL found in *QGpc.huj.uh-5B.2* between Zavitan (WEW), CS (*T. aestivum*), and Svevo (*T. turgidum*) was conducted to better understand structural variations, such as rearrangements, duplications, and inversions (Fig. 6).

Notably, the microcollinearity tool developed by (Chen et al. 2020) uses version 1 (v1) of the Zavitan genome as a reference; nevertheless, since our previous analyses used Zavitan v.2, we first made sure that the order of our target genes in the analyzed sub-QTL was similar in these two versions. The homology and microcollinearity of the 13 candidate genes showed some differences between the three species and included insertions, inversions, deletions, and translocations. Homologous genes observed in our analysis may or may not exhibit collinearity, depending on the evolutionary events that occurred after their divergence. We found three genes with no microcollinearity or homology to WEW, Svevo, or CS (Fig. 6): one gene, TRIDC5BG005570, was not annotated and was included in the 1.29 Mb segment of WEW; the second gene (TRIDC5BG005710) encodes *SYP132*, and had a homolog in *T. aestivum*, but in a different location (38.35 Mb), and the third gene (TRIDC5BG005760), which is not annotated, was homologous to both species but found in different locations, in the two genotypes (38.51 Mb in CS, and in Svevo 51.18 Mb in different region).

### Gene expression under N deficiency tested by qPCR

**Fig. 7:**
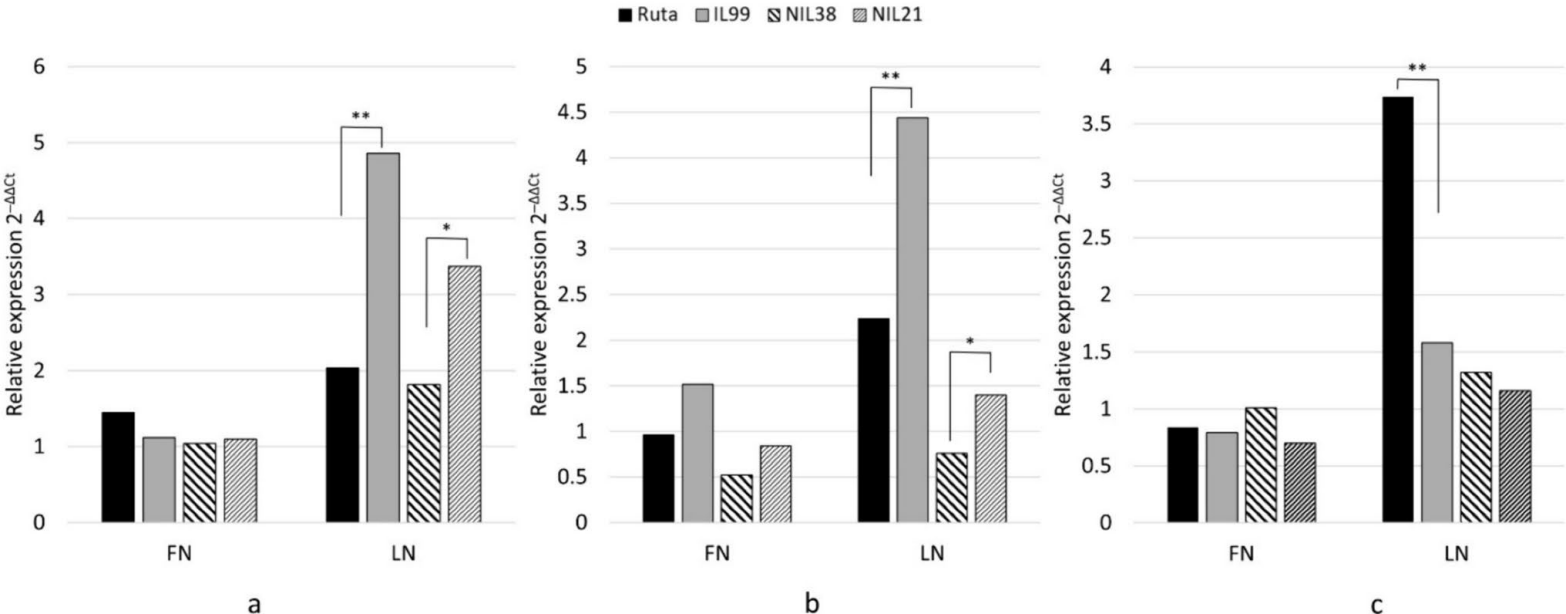
Relative expression of three candidate genes under LN and FN in IL99 and Ruta, NIL21 and NIL38. Expression was assessed by qPCR and was monitored in 14-day seedlings grown in semi-hydroponics system in a nutrient solution under FN (1.0 mM (NH_4_)_2_SO_4_ and 1.0 mM KNO_3_) and LN (0.1 mM (NH_4_)_2_SO_4_ and 0.1 mM KNO_3_). The candidate genes used: **a** XM_044536089 (*UREIDE PERMEASE TRANSPORTER*; *UPS1*), **b** XM_044530792 (*AUXIN RESPONSE FACTOR 6*; *ARF6*), **c** XM_044530780 (*NRT1/PTR FAMILY 2.11 NITROGEN TRANSPORTER*; *NRT2.11*). Ubiquitin was used as the housekeeping control (n = 3; *p* < 0.05); **significant difference between Ruta and IL99 at *p* < 0.05; *significant difference between NIL38 and NIL21 at *p* < 0.05

The expression patterns of seven genes were compared between Ruta and IL99, and between NIL21 and NIL38 under FN vs. LN using qPCR. The NILs were selected from the set of 26 NILs segregating for LN tolerance and KASP markers, which were used for fine mapping (Fig. 4). Genotyping showed that NIL21 (tolerant) and NIL38 (susceptible) have opposite QTL intervals derived from WEW. The genes for qPCR were selected based on their location along the QTL interval: *UPS1* is an N transporter that resides within the fine-mapped 1.29 Mb QTL (Table 1), and six genes are mapped along the full QTL: *auxin response factor 6* (*ARF6*), *NRT1/PTR FAMILY 2.11 nitrogen transporter (NRT)*, *ent-kaurenoic acid oxidase 1 (EKAO1*), *SEC1 family transport protein SLY1* (*SEC1*), *WD40 repeat-like protein (WD40*), and *9-cis-epoxycarotenoid dioxygenase (NCED)*. The qPCR results showed that six of the seven genes were upregulated in response to LN, three of them were differentially expressed between IL99 and Ruta: *UPS1* showed higher up-regulation in IL99, with a 2.39-fold increase (*p* < 0.05) as compared to Ruta, and a 1.85-fold increase (*p* < 0.05) in NIL21 that shows tolerance to N stress (IL99 type), compared to NIL38 (Ruta type) (Fig. 7a). *ARF6* showed a 49.5% increase in IL99 compared to Ruta, and 45.7% in NIL21 versus NIL38. The N transporter *NRT* showed a higher up-regulation of 2.36-fold in Ruta (*p* < 0.05) compared to IL99, and no significant differences were found between the NILs. Three genes that were upregulated in response to LN, i.e., *EKAO1*, *SEC1*, *WD40*, but exhibited no differences in expression between the IL99 and Ruta, or between the NILs (Table S8). *NCED*, which is located at the upper end of the QTL, was not differentially expressed between LN and FN in Ruta and IL99 and was downregulated in both NILs in response to LN.

Many studies show that NILs for specific traits or even transformants tested by RNAseq show different expressions of thousands of genes although they differ in only a small part of the genome (Xiao et al. 2016). Furthermore, genes showing higher regulation under stress can suggest that they may have a causative effect on the trait of interest but not necessarily (Peredo and Cardon 2020). Nevertheless, *UPS1* stands out as a primary candidate gene for further study for several reasons: its location within the 1.29 Mb QTL, its higher expression under LN in IL99 and NIL21 compared to Ruta and NIL38 (indicating a link between expression and LN tolerance), and recent literature highlighting roles in N transport and N stress tolerance.

## Discussion

In the current study, we describe the identification of candidate genes associated with N-deficiency tolerance conferred by QTL *QGpc.huj.uh-5B.2* that was introgressed from WEW following the “*durum* as a bridge” approach (Klymiuk et al. 2019; Merchuk-Ovnat et al. 2016; 2017). Advanced genomic tools were used to validate the efficient introgression of the QTL by optimizing introgression size, and reducing heterozygosity and linkage drag from other chromosomes of WEW and *T. durum* previous parents. Since the QTL was identified under contrasting water availability conditions, showing a higher proportion of PEV (13%) under water-limited conditions, compared to 7% under well-watered conditions (Peleg et al. 2009; Fatiukha et al. 2020), we hypothesized that water stress may have reduced N availability  for uptake. A study on the influence of severe water stress on GPC shows variations among a large collection of landraces and wheat cultivars, with G × E interactions. The study showed that GPC increased by an average of 30% under severe water stress (Elbasyoni et al. 2018). However, this study did not consider the influence of water stress severity on N availability as a potential cause for this observation. On the other hand Ravier et al. (2017) studied the influence of N deficiencies across different wheat cultivars in 18 environments, including variations drought, growth stages, fertilizer amounts, and cultivars. They concluded that a non-detrimental N deficiency at an early vegetative stage could be tolerated, and might even enhance high yield, GPC, and NUE, depending on the cultivar. In our experiment at the Upper Galilee, IL99 had a higher GPC than Ruta, that was not negatively correlated with GY. N deficiency occurred in this field due to low N-fertilizer application and an intense N depletion by heavy rains, suggesting that N stress contributed to the higher GPC in IL99 compared to Ruta. In comparison, GPC was unchanged under both water-limited and well-watered conditions when grown with adequate N-application. In the Reim field experiment, although GPC was not different in IL99 compared to Ruta, there was a significant difference (*p* < *0.05*) in GPD between the average normalized residues of Ruta and IL99. IL99 showed a positive GPD value, indicating a higher GPC than expected based on its GY. Altogether, our field results validated high GPC under severe N stress conditions in IL 99 that was conferred by the WEW QTL, possibly by a pleiotropic effect (Fan et al. 2019). To apply a severe N stress per se*,* we developed a robust semi-hydroponic system that confirmed IL99’s tolerance to N deficiency, based on leaf growth parameters as indicators for tolerance.

Fine mapping integrated the segregation of parental haplotypes with phenotype of the response to N-deficiency stress among recombinants. We delimited the QTL of 28.28 to–1.29 Mb, which was located at the center of *QGpc.huj.uh-5B.2.* This 1.29 Mb interval was shared among N-deficiency tolerance NILs, which all had the WEW haplotype. Of the 13 high-confidence genes found in this region, 11 had GO and known functions of two main categories, N-stress-related genes (including N transporters and N stress), and abiotic stress response. These genes or their combinations can be regarded as candidate genes for improving N-stress tolerance in wheat and may improve NUE and GPC or yield under severe N-deficient environments. Microcollinearity analysis of this QTL region between WEW, *durum* and bread wheat cultivars, detected collinear genes as well as structural variations. Similar findings were reported by (Maccaferri et al. 2019) who compared whole-genome sequences between WEW and *T. durum* var. Svevo.

*The following N-associated genes were also linked with abiotic stress* (Table 1)*:* *UPS1* (TRIDC5BG005550), is involved in the long-distance transport of allantoin and purine nucleotide degradation products in N-fixing and nitrate-feeding legumes (Tegeder and Perchlik 2018; Thu et al. 2020; Kaur et al. 2022; Wang et al. 2022). Furthermore, stress-regulated purine gene transcription was linked to allantoin changes that improved N utilization in plants (Tegeder and Perchlik 2018; Casartelli et al. 2019). Studies showed that allantoin is a vital N source in wheat (Casartelli et al. 2019), *Arabidopsis* (Nourimand and Todd 2019), rice (Lee et al. 2018), and barley (Shabala et al. 2016). *UPS1* and *proALN* are crucial N sensors for N remobilization in rice (Redillas et al. 2019) and (Melino et al. 2022) showed that *OsUPS1* overexpression enhances growth under low N. Meng et al. (2023) recently confirmed the roles of *TaUPS1* and *TaUPS2.1* in wheat, and Li et al. (2024) found that under sufficient N supply, there is a significant accumulation of ureide in the roots of a high-NUE wheat cultivar and suggested that its accumulation is controlled by fine regulation of key genes involved in its metabolism. These studies demonstrated an association between ureide and high NUE under different N availability, including N stress. These associations support our qPCR results showing that the expression of *UPS1* increases under LN in IL99, to a higher level than in Ruta. A second transporter *IMPORTIN SUBUNIT BETA-1 KPNB1* (TRIDC5BG005600), a main member of transport proteins that mediates the translocation of cargo protein into the nucleus through the nuclear pore complex (Bednenko et al. 2003; Palma et al. 2019). (Bonnot et al. 2017) showed that the response of the grain sub-proteome to N and sulfur supply in the diploid wheat *Triticum monococcum* ssp*. monococcum* is regulated by the *α- and β-subunits of importin (KPNB1)*. *Z-ISO* (TRIDC5BG005530) is known to be involved in antioxidant metabolism and abiotic stress in wheat (Cui et al. 2018) and is related to the *NITRITE AND NITRIC OXIDE REDUCTASE U* gene (Chen et al. 2010). Z-ISO accumulation varies under high salinity and N stress, potentially due to the transcriptional regulation of β-carotene-biosynthesis genes at the initial exposure to stress (Park et al. 2002; Zhu et al. 2020). Limited N availability reduces carotenoid content, impacting plant metabolic activity. (Shang et al. 2018) noted that N-deficiency increases phytoene desaturase gene expression, affecting carotenoid accumulation in green algae. The *serine protease HtrA* (TRIDC5BG005630) is known for proteolytic activity and involvement in protein quality control and degradation pathways (Clausen et al. 2011). *HtrA* is involved in signaling pathways and regulatory networks, influencing gene expression and stress-responsive signaling. Under N stress, it can regulate plant homeostasis and protein patterns and interact with proteins involved in N-metabolism pathways (Foucaud-Scheunemann and Poquet 2003).

*The following genes are associated with abiotic stress:* TRIDC5BG005540 encodes a *pentatricopeptide repeat protein*; these proteins are involved in mitochondrial translation in *Arabidopsis*, and resistance to abiotic stress, including salinity, drought, and cold, without negative affecting plant development (Uyttewaal et al. 2008; Jiang et al. 2015). The *HKMT protein* (TRIDC5BG005580) influences chromatin and DNA methylation under stress. Its direct link to the N-stress response is unclear, but it is hypothesized that HKMT-mediated chromatin changes help plants adapt through epigenetic regulation of N-sensitive genes (Perdiguero et al. 2009; Palma et al. 2010). The TRIDC5BG005710 gene encodes *SYP132*. This gene is involved in signaling, responses to abiotic stress and pathogens, cytokinesis, and gravitropism, and regulates protein transport during immune signaling (Surpin and Raikhel 2004; Pratelli et al. 2004; Carter et al. 2004; He et al. 2021). *SYP132* mediates tip-focused membrane trafficking for root hair-tip growth, demonstrating its importance in the development and function of root hairs in *Arabidopsis* (Ichikawa et al. 2014). Its involvement with gravitropism and root tip growth is interesting since a previous study in durum wheat identified a QTL for root spread angle in the vicinity of our QTL (Sanguineti et al. 2007). Two other genes (TRIDC5BG005560 and TRIDC5BG005770) were associated with abiotic stress. The first activates *cold-regulated protein* (*COR*) for plant adaptation to cold and drought stress tolerance (Guo et al. 2019). The second, TRIDC5BG005770, is in the *MHO* superfamily, it responds to abiotic stress-triggered abscisic acid and ethylene signaling. *MHO* enzymes may impact reactive oxygen species clearance or redox regulation in plants under stress (Wang et al. 2023). TRIDC5BG005520 belongs to the *p21-activated kinases* (*PAKs*), a family of protein kinases that play essential roles in various cellular processes, including signal transduction, cytoskeletal organization, and plant stress responses (Bokoch 2003). PAKs can be involved in nutrient signaling and uptake processes (Sun et al. 2022; Tian et al. 2023).

## Conclusions and prospects

N deficiency may occur in the soil at the young growth stage because of a lack of pre-sowing N fertilization, leaching by heavy rains, drought, or other abiotic stresses (e.g., unfavorable pH, ionic stress, soil salinity, or acidity), thereby, negatively affecting grain yield and quality. The adaptation to N-deficiency is involved in plant developmental processes, including morphological modifications of shoot and root-system architecture controlled by large changes in gene expression (Khan et al. 2017; Ru et al. 2023; Munns and Millar 2023). Fine mapping of the *QGpc.huj.uh-5B.2* enabled us to identify candidate genes for LN tolerance, which may improve NUtE and GPC in N-deficient environments. Further studies using CRISPR–Cas9 should functionally characterize these genes, and may allow us to determine if and which one of the genes has a major role in the response to LN.

Cultivars that are tolerant to N deficiency can grow under lower levels of N fertilizer and improve NUE (Vishnukiran et al. 2020). Such cultivars are especially suitable for soil that tends to lose N at the plant establishment, vegetative growth, or for organic farming. Furthermore, considering the low variability of NUE in cultivars, currently, the best way for crop improvement is the introgression of traits from the available wild progenitor gene pool (Munns and Millar 2023). Hence, the results of the current study demonstrate that WEW, which is known as an important source for biotic and abiotic stress improvement in wheat, can also serve as a rich source of novel genes for N-deficiency tolerance as a mechanism to increase GPC under low-N environments, and can contribute to sustainable agriculture.

### Supplementary Information

Below is the link to the electronic supplementary material.

Supplementary file1 (XLSX 660 kb)

## Data Availability

All data supporting the reported results are included within the article or in the Supplementary Information.
